# miR-122 promotes diabetic retinopathy through targeting TIMP3

**DOI:** 10.1080/19768354.2020.1816580

**Published:** 2020-09-10

**Authors:** Mingliang Wang, Huifen Zheng, Xianbo Zhou, Jiwei Zhang, Guanghui Shao

**Affiliations:** a Department of Ophthalmology, Hangzhou Lin’an District People’s Hospital, Hangzhou City, People’s Republic of China; b Department of Ophthalmology, Dongying Shengli Hospital of Traditional Chinese Medicine, Dongying City, People’s Republic of China

**Keywords:** Diabetic retinopathy, miR-122, TIMP3, cell viability, cell apoptosis

## Abstract

Diabetic retinopathy (DR) is a primary complication of diabetes mellitus. DR can cause severe vision loss for patients. miR-122 is elevated in DR patients, while its role in DR is unclear. Hence, the purpose of this study was to analyze the effect of miR-122 on the function of high glucose-induced REC cells and the underlying molecular mechanisms. In this study, our results revealed that miR-122 was up-regulated in high glucose-induced human retinal pigment epithelial cells (ARPE-19). High glucose decreased the cell viability of ARPE-19 cells, which was then restored by miR-122 knockdown. In addition, miR-122 knockdown suppressed apoptosis of high glucose-induced ARPE-19 cells. High glucose also inhibited B-cell lymphoma-2 (Bcl-2) level and increased cleaved caspase-3 level in ARPE-19 cells, which were reversed by miR-122 knockdown. Tissue inhibitor of metalloproteinases-3 (TIMP3) was a direct target of miR-122. TIMP3 was decreased in high glucose-induced ARPE-19 cells, and the decrease was abrogated by miR-122 knockdown. In addition, the effects of miR-122 overexpression in cell viability and apoptosis of high glucose-induced ARPE-19 were abolished by overexpression of TIMP3. In conclusion, the effect and mechanism of miR-122 on high glucose-induced ARPE-19 cells were demonstrated for the first time. miR-122 promoted diabetic retinopathy through targeting TIMP3, making miR-122 a promising target for diabetic retinopathy therapy.

## Introduction

Diabetes mellitus (DM) is a complex metabolic disease with high morbidity worldwide, accompanied by various complications (Schmidt 2018). Diabetic retinopathy (DR) is a primary complication of DM patients. DR can cause severe vision loss for patients (Stewart 2016). Therefore, DR is considered to be a significant cause of vision loss (Bourne et al. 2014). Although the therapeutic strategies for DR patients have improved in recent years, the prognosis remains poor (Stewart 2016). Studies showed that DR was mainly caused by hyperglycemia (Wang et al. 2016; Xiao and Liu 2019). Besides, retinal endothelial cell (REC) was proved to dysfunction after high glucose induction (Wu et al. 2016). However, the regulation mechanism of high glucose on the REC function is still unclear. Therefore, it is essential to investigate the mechanism of high glucose on REC function.

MicroRNAs (miRNAs) are one kind of non-coding RNAs composed of 21–23 nucleotides (Felekkis et al. 2010). MiRNAs function as a post-transcriptional regulator to modulate gene expression via targeting to the 3′-untranslated region (3′-UTR) of the mRNA (Bartel 2009; Hu et al. 2019). Accumulating evidence reported that miRNAs could modulate DR progression though modulating cellular biological processes such as cell proliferation, migration, and apoptosis (Martinez and Peplow 2019; Shafabakhsh et al. 2019). For example, miR-219-5p modified DR development by modulating human retinal pigment epithelial (RPE) cells apoptosis through regulation of LRH-1/Wnt/*β*-Catenin signaling pathway (Zhao et al. 2018). miR-152 could suppress angiogenesis caused by high glucose in RPE cells via targeting LIN28B (Fu and Ou 2020). As an important member of miRNAs, miR-122 was proved to participate in the regulation of diseases such as gastric cancer (Rao et al. 2017), liver cancer (Coulouarn et al. 2009), and ischemic stroke (Guo et al. 2018). Furthermore, miR-122 was demonstrated to have aberrant expression in the serum of DR patients (Pastukh et al. 2019). miR-122 levels increased with the severity of retinopathy (Pastukh et al. 2019). Hence, miR-122 may have a role in the pathogenesis of DR.

Thus, the purpose of the study was to illustrate the effect of miR-122 in the function of high glucose-induced REC cells and the underlying molecular mechanisms, which may be beneficial for exploring the potential therapeutic target for DR patients.

## Materials and methods

### Cell culture

Human retinal pigment epithelial cells ARPE-19 were acquired from the American Type Culture Collection (ATCC). ARPE-19 cells were cultured in DMEM medium plus 10% fetal bovine serum (FBS) (Gibco, Carlsbad, CA, USA) at 37 °C, 5% CO_2_ cell incubator. In indicated experiments, high glucose (25 mM) was used to stimulate ARPE-19 cells, while the control ARPE-19 cells were treated with 5 mM glucose for 24 h.

### Cell transfection

The miR-122 mimic, negative control mimic (NC mimic), negative control inhibitor (NC inh), miR-122 inhibitor (miR-122 inh), and TIMP3 overexpression plasmid were obtained from Ribobio (Ribobio, Guangzhou, China). ARPE-19 cells were plated onto 6-wells plates and cultured for 24 h. Afterward, the ARPE-19 cells in the logarithmic phase were transfected with NC inh, miR-122 inh, TIMP3 overexpression plasmid utilizing Lipofectamine 3000 (Invitrogen, Carlsbad, CA, USA) following the manufacturer’s instructions. After 48 h of transfection, ARPE-19 cells were utilized for subsequent assays.

### Quantitative real-time polymerase chain reaction (qRT-PCR)

RNA was isolated from ARPE-19 cells using Trizol (Sigma, St Louis, MO, USA), followed by reverse transcription using Mir-X miRNA First-Strand Synthesis Kit (Takara, Beijing, China). The generated cDNA was applied to perform qPCR assay using Mir-X miRNA qRT-PCR TB Green Kit (Takara, Beijing, China). The sequences of primers were as below: miR-122: F: 5′- TCTTCCTGGAATTCAAGCCTTT-3′, R: 5′- AGTGGGCCTAGTGCTGGAAA -3′ (Wang et al. 2012). *U6* was used as the control gene. 2^−ΔΔCt^ was used to analyze the relative RNA expression.

### MTT assay

ARPE-19 cells were plated onto 96-wells plates and cultured for 24 h. The cells were incubated with 20 μl MTT (Sigma, St. Louis, MO, USA) and incubated at 37°C. After 4 h, dissolving crystallization was conducted by mixing 100 μl DMSO to cells. Afterwards, the absorbance was determined using the microplate reader at 490 nm.

### Flow cytometry

ARPE-19 cells were harvested and washed in PBS, and centrifuged for 5 min at 1000 rpm, following by resuspension in binding buffer. Afterward, cells were dyed with 5 μl of Annexin V-FITC and 10 μl of PI (BD, San Jose, CA, USA) in the dark for 15 min. The proportion of apoptosis was measured on the flow cytometry (BD, San Jose, CA, USA).

### Western blot

The proteins in ARPE-19 cells were isolated using RIPA (Sigma, St. Louis, MO, USA), and then subjected to SDS-PAGE gels. Afterwards, the lysates were transferred onto the PVDF membrane, following by probing with anti-TIMP3 (1:500), B-cell lymphoma-2 (Bcl2) (1:500), cleaved caspase-3 (1:500), and GAPDH (1:500) antibodies overnight at 4 °C after the membrane was blocked with 4% skim milk for 1 h. Next, the membrane was treated with secondary antibody (Abcam, Cambridge, UK). The blots were then imaged utilizing ECL Western Blotting Substrate (Thermo Fisher Scientific, Waltham, MA, USA).

### Luciferase assay

The miR-122 mimic, NC mimic, wild-type, and mutant 3′-UTR of TIMP3 were obtained from Ribobio (Ribobio, Guangzhou, China). Two types 3′-UTR of TIMP3 were inserted into the pGL3 vector (Promega, Madison, WI, USA), respectively. The pGL3 vector with wild-type 3′-UTR of TIMP3 or mutant 3′-UTR of TIMP3, miR-122 mimic, or NC mimic were transfected into ARPE-19 cells, respectively. After 48 h, the microplate reader determined the luciferase activity by normalizing to Renilla luciferase activity.

## Statistical analysis

Data statistical analysis was carried out using SPSS Statistics 22.0 (Chicago, IL, USA). Data were presented as mean ± standard deviation (SD). The group differences were determined using one-way ANOVA and Student’s *t*-test. *p* < 0.05 was regarded as statistically significant.

## Results

### miR-122 knockdown enhanced cell viability of high glucose-induced ARPE-19 cells

**Figure 1.**
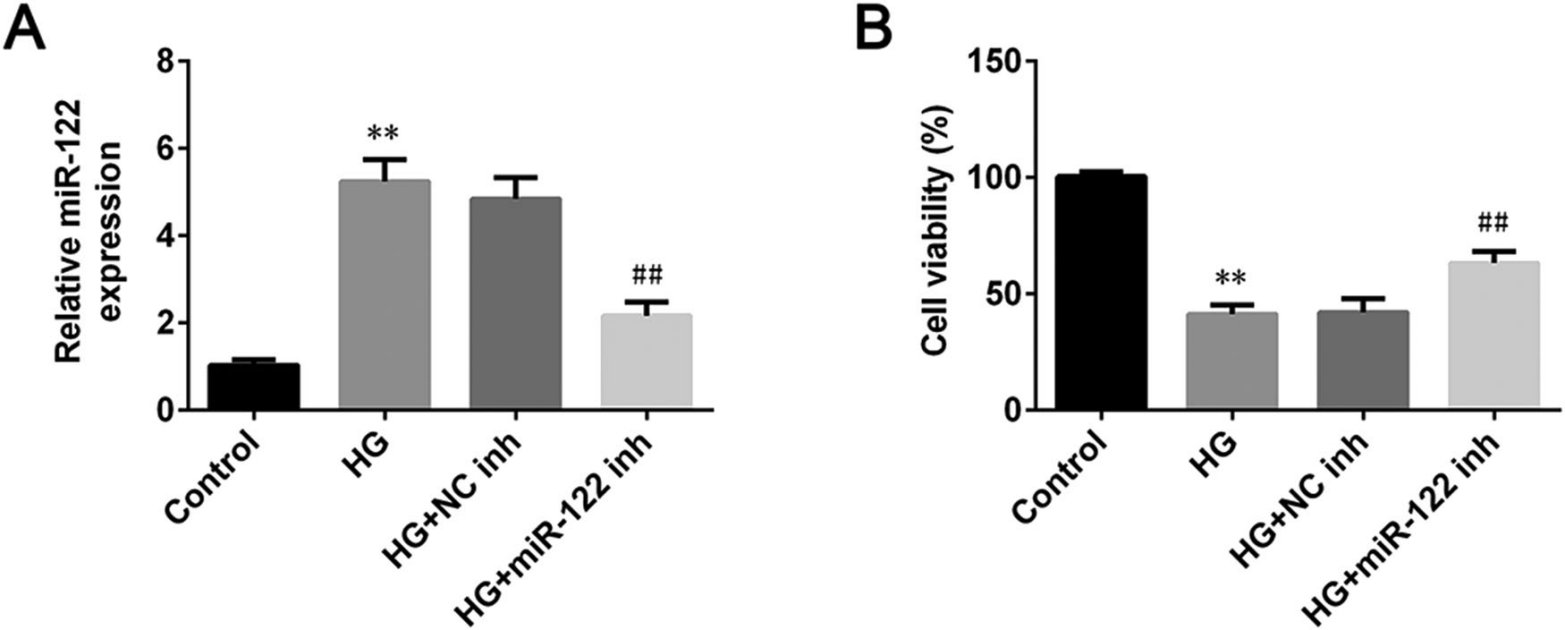
**miR-122 knockdown enhanced cell viability of high glucose-induced ARPE-19 cells (A)** qRT-PCR was used to determine the expression of miR-122 in ARPE-19 cells treated with high glucose and transfected with the miR-122 inhibitor (**B)** The cell viability of ARPE-19 cells treated with high glucose and transfected with miR-122 inhibitor was measured by MTT assay. **: *p* < 0.01. ##: *p* < 0.01.

To investigate the influence of miR-122 on the pathogenesis of DR, the expression of miR-122 in ARPE-19 cells stimulated wigh high glucose was measured by qRT-PCR. The miR-122 was up-regulated in high glucose-induced ARPE-19 cells (*p* < 0.01, Figure 1(A)). After transfection of miR-122 inhibitor, the expression of miR-122 significantly decreased in the high glucose-induced ARPE-19 cells (*p* < 0.01, Figure 1(A)). Besides, the cell viability of ARPE-19 was reduced after treated with high glucose (*p* < 0.01), which was reversed by miR-122 knockdown (*p* < 0.01, Figure 1(B)). Collectively, miR-122 knockdown increased the viability of high glucose-induced ARPE-19 cells.

### miR-122 knockdown suppressed apoptosis of high glucose-induced ARPE-19 cells

**Figure 2.**
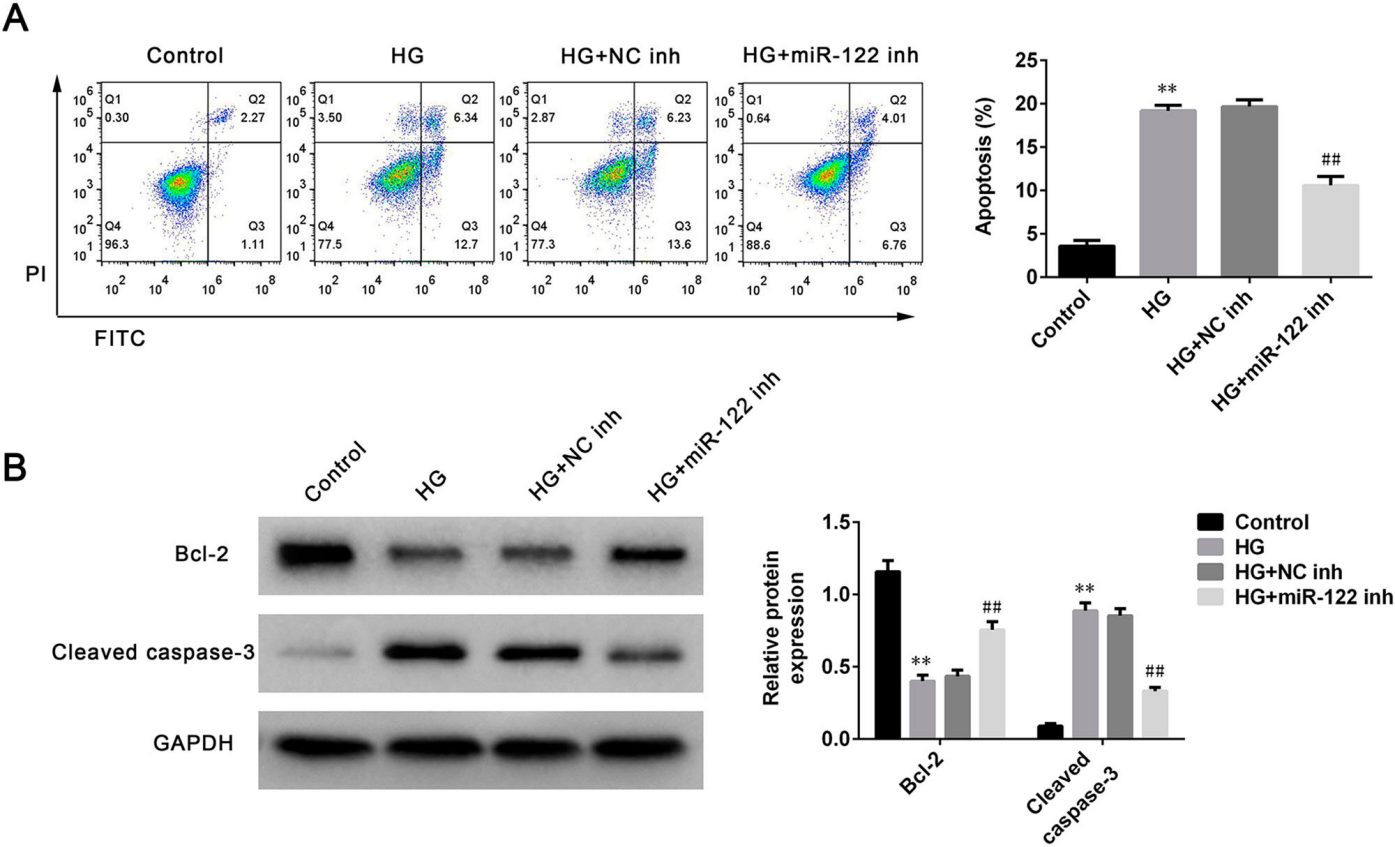
**miR-122 knockdown suppressed cell apoptosis of high glucose-induced ARPE-19 cells (A)** Flow cytometry was used to determine apoptosis of ARPE-19 cells treated with high glucose and transfected with the miR-122 inhibitor (**B)** Western blot was performed to measure the levels of Bcl-2 and cleaved caspase-3 in ARPE-19 cells treated with high glucose and transfected with the miR-122 inhibitor. **: *p* < 0.01. ##: *p* < 0.01.

To further study the function of miR-122 on DR progression, the apoptosis of ARPE-19 cells stimulated by high glucose was determined after miR-122 knockdown. High glucose robustly induced apoptosis proportion of ARPE-19 cells (*p* < 0.01), which was reversed by miR-122 knockdown (*p* < 0.01, Figure 2(A)). Next, the levels of apoptosis-related proteins in ARPE-19 cells stimulated by high glucose and transfected with miR-122 inhibitor were determined. The level of Bcl-2 was significantly decreased in high glucose-induced ARPE-19 cells (*p* < 0.01, Figure 2(B)). However, the miR-122 inhibitor greatly enhanced the level of Bcl-2 in ARPE-19 cells stimulated by high glucose compared to the control inhibitor (*p* < 0.01, Figure 2(B)). Furthermore, the level of cleaved caspase-3 was increased in ARPE-19 cells treated with high glucose (*p* < 0.01), which was reversed by miR-122 knockdown (*p* < 0.01, Figure 2(B)). Therefore, miR-122 knockdown suppressed apoptosis of high glucose-induced ARPE-19 cells.

### TIMP3 was a direct target of miR-122

**Figure 3.**
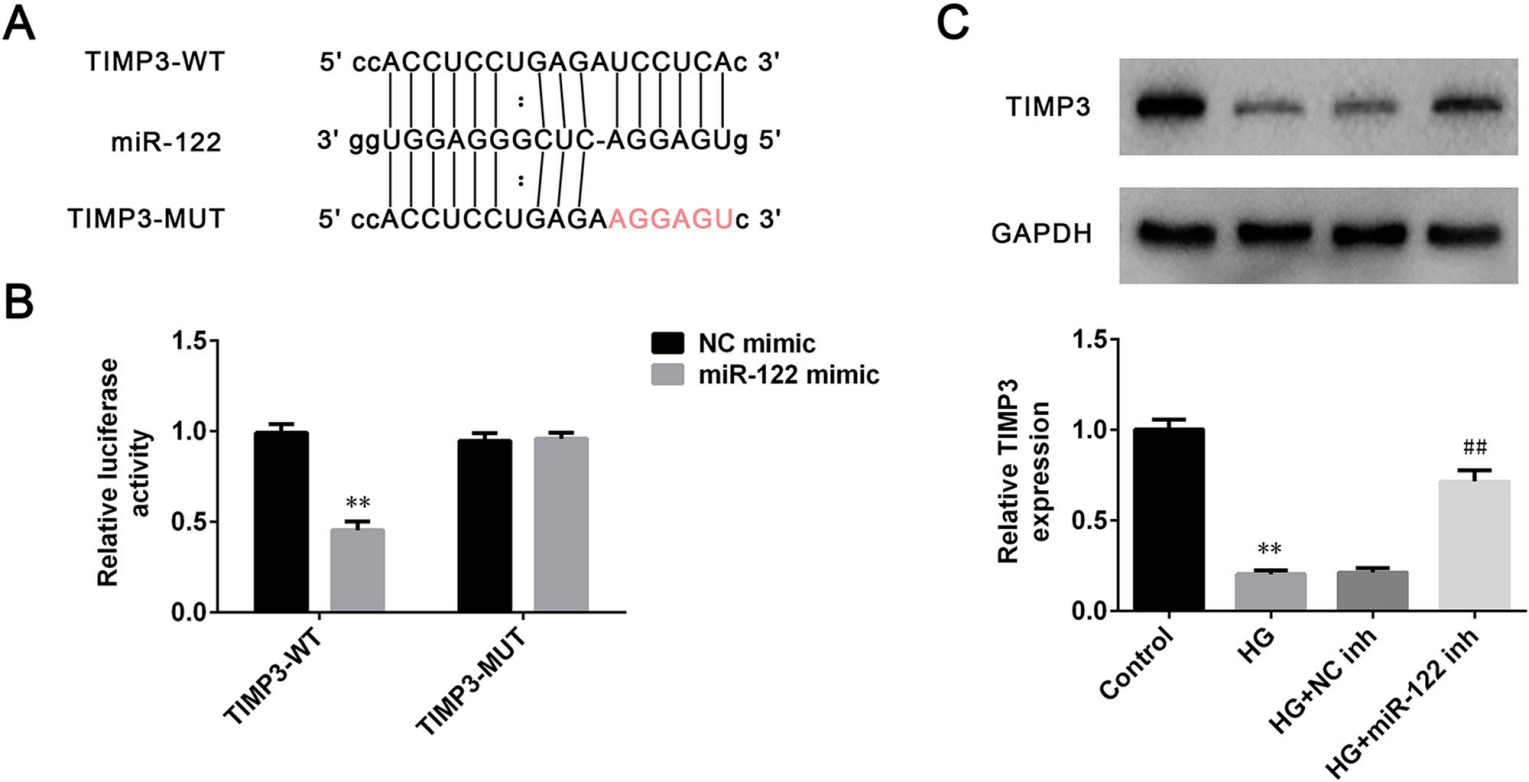
**TIMP3 was a direct target of miR-122 (A)** The miR-122 wild-type and mutant binding sites in the 3′-UTR of TIMP3 were shown (**B)** Luciferase assay was conducted to verify the relationship between miR-122 and TIMP3 (**C)** The level of TIMP3 in ARPE-19 cells treated with high glucose and transfected with miR-122 inhibitor was determined by western blot. **: *p* < 0.01. ##: *p* < 0.01.

MiRNAs were demonstrated to participate in the regulation of diseases generally via binding to the target genes. Therefore, the target gene of miR-122 was explored through screening the starbase (http://starbase.sysu.edu.cn/). Results demonstrated that TIMP3 was a potential target for miR-122, and the putative targeting sequences of miR-122 and TIMP3 were presented in Figure 3(A). To further confirm whether TIMP3 was the target of miR-122, the luciferase assay was conducted in ARPE-19 cells. Results revealed that miR-122 overexpression robustly reduced the relative luciferase activity of ARPE-19 cells transfected with wild-type 3′-UTR of TIMP3 (*p* < 0.01), while had no significant effects on the relative luciferase activity of ARPE-19 cells transfected with the mutant 3′-UTR of TIMP3 (Figure 3(B)). In addition, the level of TIMP3 was significantly inhibited in high glucose-induced ARPE-19 cells (*p* < 0.01), which was abrogated by miR-122 knockdown (*p* < 0.01, Figure 3(C)). Thus, TIMP3 was a direct target of miR-122.

### miR-122 inhibited cell viability and promoted apoptosis of high glucose-induced ARPE-19 cells by regulating TIMP3

**Figure 4.**
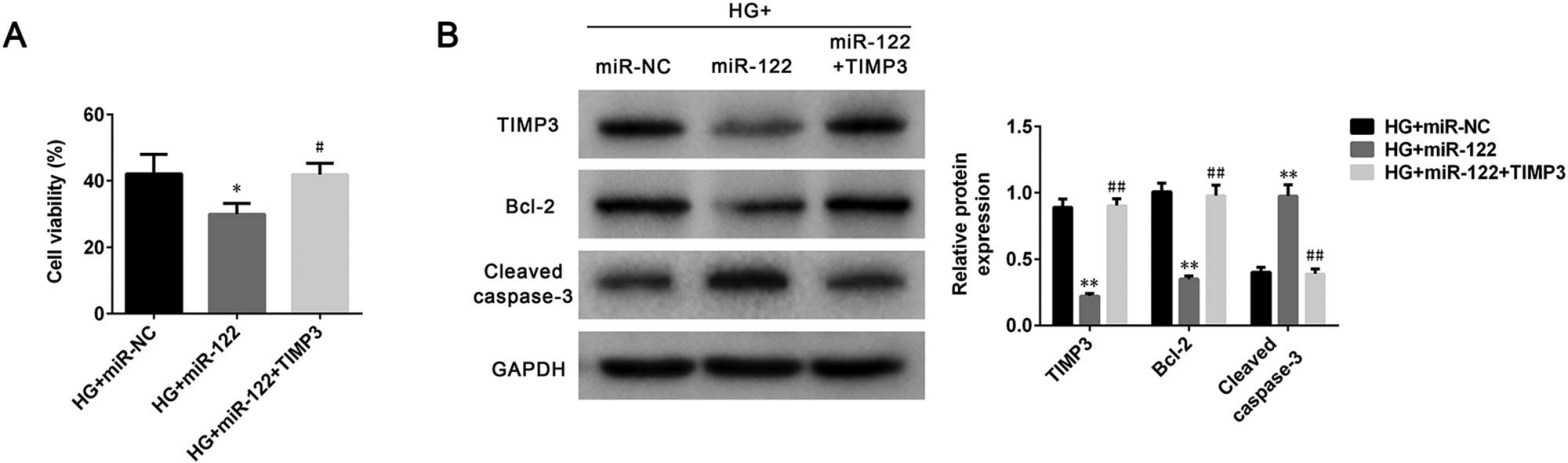
**miR-122 promoted cell apoptosis of high glucose-induced ARPE-19 cells by regulating TIMP3 (A)** The cell viability of ARPE-19 cells treated with high glucose and transfected with miR-122 mimic or TIMP3 overexpression plasmid was measured by MTT assay (**B)** The levels of TIMP3, Bcl-2, and cleaved caspase-3 in ARPE-19 cells treated with high glucose and transfected with the miR-122 mimic, or TIMP3 overexpression plasmid were determined using western blot. *: *p* < 0.05. **: *p* < 0.01. #: *p* < 0.05. ##: *p* < 0.01.

To study the modulation mechanism of miR-122 on DR progression, cell viability and apoptosis were determined after ARPE-19 cells were treated with high glucose and transfected with miR-122 mimic or TIMP3 overexpression plasmid. miR-122 overexpression decreased cell viability of high glucose-induced ARPE-19 cells (*p* < 0.05), which was abolished by TIMP3 overexpression (*p* < 0.05, Figure 4(A)). The level of TIMP3 was inhibited by miR-122 overexpression in high glucose-induced ARPE-19 cells (*p* < 0.01), which was abrogated by TIMP3 overexpression (*p* < 0.01, Figure 4(B)). Furthermore, miR-122 overexpression also decreased the level of Bcl-2 (*p* < 0.01), which was reversed by TIMP3 overexpression in high glucose-induced ARPE-19 cells (*p* < 0.01, Figure 4(B)). Moreover, miR-122 overexpression promoted the level of cleaved caspase-3 in high glucose-induced ARPE-19 cells (*p* < 0.01), which was abolished by TIMP3 overexpression (*p* < 0.01, Figure 4(B)). Together these results suggested miR-122 inhibited cell viability and promoted cell apoptosis of high glucose-induced ARPE-19 cells by regulating the expression of TIMP3.

## Discussion

Diabetic retinopathy (DR) is a primary complication of diabetes mellitus patients (Stewart 2016). In consideration of the severity of DR on the vision of patients and the limitation of therapeutic strategies for patients (Bourne et al. 2014; Stewart 2016), it is critical to search the potential target for DR. Hyperglycemia is the primary cause for DR, and it can induce the death of REC cells (Wang et al. 2016). However, the regulation mechanism of high glucose on REC cells is not fully established.

MiRNAs were proved to involve in the regulation of DR (Martinez and Peplow 2019). Martinez and Peplow (2019) reported that miRNAs were dysregulated in DR patients’ vitreous humor, indicating that miRNA might be biomarkers of DR. Besides, some miRNAs participated in the regulation of REC function (Jiang et al. 2016; Thounaojam et al. 2019). For example, miR-34a regulated stress-associated premature senescence in high glucose-induced REC cells (Thounaojam et al. 2019). miR-122 was elevated in the serum of DR patients (Pastukh et al. 2019), suggesting that miR-122 might regulate REC function and modulate DR progression. Therefore, the roles of miR-122 on high glucose-induced REC function were studied. The results in the current study revealed that the expression of miR-122 was up-regulated in high glucose-induced ARPE-19 cells. This result was in agreement with the findings obtained by Pastukh et al. (2019). Besides, we showed that high glucose suppressed cell viability and promoted apoptosis of ARPE-19 cells, revealing that high glucose impaired the cell function of ARPE-19 cells. The injury of high glucose on REC cells has been reported in some articles (Jiang et al. 2016). For example, Jiang et al. (2016) proved that high glucose affected cell proliferation ability of REC cells. Xiao and Liu (2019) found that high glucose promoted inflammation and apoptosis of ARPE-19 cells. Furthermore, this study revealed that the influences of high-glucose on cell viability and apoptosis were reversed by miR-122 knockdown. In other words, miR-122 could regulate the viability and apoptosis of ARPE-19 cells stimulated by high glucose.

MiRNAs usually act as diseases regulators via binding to the target genes (Felekkis et al. 2010). To explain the underlying mechanisms of miR-122 on ARPE-19 cells stimulated by high glucose, the target gene of miR-122 was explored. According to bioinformatics analysis and luciferase assay results, we found that TIMP3 was the target of miR-122. Besides, the level of TIMP3 was negatively modulated by miR-122 in high glucose-induced ARPE-19 cells. TIMP3 has been found to suppress vascular leakage and leukostasis by inhibiting the expression of VEGF and TNF-α in DR (Poulaki et al. 2007). Besides, inhibition of TIMP3 protected the proliferative retinopathies in mice by inhibiting neovascularization (Hewing et al. 2013). The previous study also showed that TIMP3 mediated the modulation effects of miR-365 on DR progression (Wang et al. 2018). In addition, overexpression of miR-221-3p facilitated the microvascular dysfunction in DR via targeting TIMP3 (Wang et al. 2020). miR-770-5p promoted podocyte apoptosis and inflammation response in DR through targeting TIMP3 (Wang and Li 2020). These evidence indicated that TIMP3 might exert vital roles in DR, and TIMP3 might mediate the regulation process of miR-122 on cell viability and apoptosis of high glucose-induced ARPE-19. Therefore, the function of TIMP3 in the modulation of miR-122 on high glucose-induced ARPE-19 was investigated. TIMP3 overexpression abolished miR-122 overexpression induced decrease in cell viability and increase in apoptosis. These results revealed that miR-122 inhibited cell viability and promoted apoptosis of high glucose-induced ARPE-19 cells by modulating TIMP3. The role of TIMP3 in cell viability and apoptosis has been reported in previous studies. TIMP3 mediated the effect of miR-142-3p on promoting cell viability and inhibiting apoptosis following sciatic nerve injury (Wu et al. 2018). In addition, cell viability of human granulosa-lutein cells was decreased after treating with TIMP3 (Rosewell et al. 2013). TIMP3 promoted apoptosis through a caspase-independent mechanism in endothelial cells (Qi and Anand-Apte 2015). Hence, we concluded that miR-122 promoted diabetic retinopathy through targeting TIMP3, and miR-122 might be a useful treatment target for DR.

## Conclusion

In the study, the effect and mechanism of miR-122 on high glucose-induced ARPE-19 cells were demonstrated for the first time. miR-122 promoted diabetic retinopathy through targeting TIMP3, making miR-122 a promising target for diabetic retinopathy therapy.

## Availability of data and materials

All data generated or analyzed during this study are included in this published article.
